# Complete Genome Sequence Analysis of a Vaccine Strain of Foot-and-Mouth Disease Virus Serotype O from Pakistan

**DOI:** 10.1128/genomeA.00958-15

**Published:** 2015-09-03

**Authors:** Muhammad Zubair Shabbir, Muhammad Munir

**Affiliations:** a Quality Operations Laboratory, University of Veterinary and Animal Sciences, Lahore, Pakistan; b The Pirbright Institute, Woking, United Kingdom

## Abstract

Sequencing and subsequent analysis of a vaccine strain of foot-and-mouth disease virus serotype O is reported here. Genomic heterogeneity in the protective epitopes (VP1 protein) of the reported strain, compared to characterized strains and available sequences from Pakistan, warrants further studies to determine vaccine-induced immunity and disease protection.

## GENOME ANNOUNCEMENT

Foot-and-mouth disease (FMD) is a highly contagious disease of cloven-hoofed animals caused by FMD virus, genus *Apthovirus*. It has a single-stranded positive-sense RNA genome of approximately 8.3 kb. The capsid is composed of 60 copies each of four different structural proteins (VP1 through VP4) (1, 2). With the potential to mutate rapidly, it exists in seven distinct antigenic and immunologic serotypes: Euroasiatic (O, A, C, and Asia-1) and South African Territories (SAT-1, SAT-2, and SAT-3) (3). Serotype O is pandemic globally and, based upon 15% nucleotide differences in the VP1 coding sequence, strains are grouped into eight topotypes (4). The O-PanAsia II^ANT-10^ strain of serotype O belonging to the Middle East–South Asia (ME-SA) topotype is known to have caused more disease outbreaks in Pakistan than serotype A and Asia-1 (5).

The reported virus was isolated from an outbreak in a bovine herd originating from the Narowal district of Punjab. While the previous antigen capture enzyme-linked immunosorbent assay (ELISA) and VP-1 gene-based analysis revealed the isolate as serotype O, the complete genetic nature remained elusive. Here, we describe the whole genetic picture of this vaccine strain, using next generation sequencing platform (Illumina NextSeq500). Using the KAPA Stranded RNA-Seq library preparation kit, libraries were prepared from cell culture-derived genomic RNA. The workflow consisted of double-stranded cDNA generation using a mixture of random and poly(T) priming, fragmentation of double-stranded cDNA, end repair to generate blunt ends, A-tailing, adaptor ligation, and PCR amplification. A quality check of the data was done on Illumina SAV, and demultiplexing was performed with Illumina CASAVA version 1.8.2. Reads were mapped to the corresponding virus genome using Bowtie2 version 2.1.0. PRICE was used to assemble the complete genome and used in the phylogenetics and molecular analysis.

The complete genome of the vaccine strain is 8,157 nucleotides (nt) in length, including a 1,051-nt 5′ untranslated region (UTR), a 6,999-nt open reading frame (ORF) encoded for a polypeptide 2,332 amino acids long, and a 107-nt 3′ UTR with a 14-nt poly(A) tail. The polypeptide ORF spans between 1,052 and 8,050 nt, a characteristic for serotype O. Initial BLAST analysis of the full-genome sequence indicated clustering of strains belonging to serotype O. The full-length genome (8,157 nt) shared identities of 93%, 92%, and 91% with the isolates from India (AY593828), Turkey (AY593823), and Pakistan (GU384682 and GU384683), respectively. Interestingly, comparison of the only VP-1 gene (639 bp) of the study isolate to that of sequences reported from 2002 to date from Pakistan revealed a higher percentage similarity for sequences reported before (88.89% to 99.37%) and during (89.10% to 90.05%) the year 2005 than those reported in the later years till now (87.67% to 89.89%). Since, over time, differences have been observed in the protective epitopes in the capsid VP1 protein, there is a need to determine the roles of these differences in terms of vaccine-induced immunity and disease protection, using serum neutralization assays and virus challenge studies, respectively.

### Nucleotide sequence accession number.

The complete genome sequence of a vaccine strain (Nari/UVAS-Pak/2005) has been submitted to GenBank under the accession number KT003716.
